# Beef Consumption Is Associated with Higher Intakes and Adequacy of Key Nutrients in Older Adults Age 60+ Years: National Health and Nutrition Examination Survey 2011–2018 Analysis

**DOI:** 10.3390/nu16111779

**Published:** 2024-06-06

**Authors:** Sanjiv Agarwal, Victor L. Fulgoni

**Affiliations:** 1 NutriScience, LLC, East Norriton, PA 19403, USA; 2 Nutrition Impact, LLC, Battle Creek, MI 49014, USA; vic3rd@aol.com

**Keywords:** fresh beef, ground beef, processed beef, protein, B vitamins, iron, zinc, choline

## Abstract

Beef is an important source of high-quality protein and several micronutrients, including iron, zinc, and B vitamins. We determined beef intake and its relationship with intakes of nutrients and their adequacy using 24 h dietary recall data from 5868 older adults. Usual intakes from foods were determined using the National Cancer Institute method, and the percent of the population below the estimated average requirement or above adequate intake was estimated. A high percentage of older adults did not meet nutrient recommendations for vitamin D (96%), choline (96%), vitamin E (84%), potassium (70%), calcium (63%), magnesium (60%), vitamin C (46%), vitamin A (39%), zinc (21%), vitamin B_6_ (19%), and folate (15%). About 68% of older adults were beef consumers with a mean intake of 56 g/day. Beef consumers had higher (*p* < 0.05) intakes of energy, protein, calcium, iron, phosphorus, selenium, sodium, zinc, thiamin, riboflavin, niacin, vitamin B_12_, and choline, and a higher (*p* < 0.05) proportion met nutrient recommendations for protein, calcium, copper, zinc, thiamin, folate, and vitamin B_12_ than non-consumers. Consumers of fresh, ground, and processed beef also had generally higher intakes and lower inadequacies of many nutrients depending on the beef type. In conclusion, older adults generally had poor nutrient adequacy from their diets, while beef consumers had higher nutrient intakes and adequacy for certain key nutrients, which are inherently generally available from beef or from foods consumed with beef.

## 1. Introduction

The population of older adults age 60+ years is continuously increasing worldwide and is projected to increase from 1 billion people in 2019 to 1.4 billion by 2030 and 2.1 billion by 2050 [1]. The population of older Americans (age 65+ years) is also projected to increase by 47% from 58 million in 2022 to 82 million by 2050 [2]. The aging process is associated with several physical, physiological, and cognitive changes in the body. Therefore, this anticipated increase in the older adult population is expected to potentially create additional healthcare and support services requirements, as 23% of the global burden of disease is attributable to health issues of older people [3].

Adequate nutrition throughout the lifespan helps prevent chronic disease and supports healthy aging. Older adults are also at greater risk of chronic diseases and health conditions related to changes in muscle and bone mass. Although they generally have lower calorie needs because of their less active lifestyle and slower metabolism, their nutrient needs are similar or even higher compared to younger adults because of changes in their body composition due to aging, less efficient ability to absorb nutrients, and lower ability to utilize many nutrients [4]. Therefore, following a healthy dietary pattern that includes nutrient-dense foods and maintaining adequate nutritional status is particularly important to this age group. However, many older adults consume less food and skip meals, resulting in suboptimal intakes of energy and key nutrients [5,6]. A significant proportion of American adults, including older adults, under-consume calcium, magnesium, zinc, vitamin A, vitamin C, vitamin D, vitamin E, vitamin K, and choline [7]. The Dietary Guidelines for Americans 2020–2025 have also indicated that older adults do not consume enough fiber, calcium, potassium, and vitamin D (“nutrients of public health concern”) and are also at increased risk for inadequate intakes of protein and vitamin B_12_ [8].

In the American diet, beef is a common food and is an important source of high-quality animal protein and several key micronutrients, including highly bioavailable iron, zinc, and B vitamins [9,10,11,12]. Lean meat (including beef) consumption as part of an overall healthy diet has been recommended by the Dietary Guidelines for Americans 2020–2025 [8]. Intake of lean beef contributed to the intake of energy and key nutrients [13] and was associated with higher intakes of protein and key micronutrients [14,15]. We recently reported that the intake of beef makes a significant contribution to daily dietary intakes of protein, B vitamins, zinc, and iron in the diet [16].

Beef and other animal foods can be a source of bioavailable nutrients commonly lacking around the world and can make important contributions to food/nutrition security. Animal food production can have environmental impacts, but it depends on local situations. Beal et al. [17] concluded animal foods “… do play important and distinct roles in achieving healthy and sustainable food systems in different contexts worldwide and will continue to do so for the foreseeable future. Efforts are needed to ensure best practices of production, curb excess consumption where high, and sustainably increase consumption where low”.

We hypothesize that since older adults have higher needs and suboptimal nutrient intakes, the intake of beef as a rich source of high-quality protein and key micronutrients would improve nutrient intakes and nutrient adequacy. Therefore, the objective of the present research was to assess the association of beef (including fresh, ground, and processed beef) intake with nutrient intake and the proportion of older adults meeting nutrient recommendations using the National Health and Nutrition Examination Survey (NHANES) data.

## 2. Materials and Methods

### 2.1. Database and Study Population

We used NHANES 2011–2018 data for the present analysis. NHANES is a continuous cross-sectional survey conducted by the National Center for Health Statistics of the Centers for Disease Control and Prevention (CDC). Data are based on a complex stratified multistage cluster sampling probability design to provide a nationally representative population to monitor food/nutrient intake and the health status of the US population [18]. We combined the data from older adults age 60+ years participating in the NHANES 2011–2012, 2013–2014, 2015–2016, and 2017–2018 cycles for the present analyses. NHANES participants were interviewed in their homes for demographic, socioeconomic, dietary (24 h dietary recall), and general health information, followed by a comprehensive health examination conducted in a mobile examination center. A detailed description of the subject recruitment, survey design, and data collection procedures is available online [18]. The total number of older adult subjects age 60+ years for this study was 5868 (representing 63.6 million older adults) after the exclusion of subjects who were missing their first or second day of 24 h dietary recall data. NHANES research protocol was approved by the National Center for Health Statistics Research Ethics Review Board, and signed written informed consent forms were collected from all participants. The present study did not require any additional approvals by institutional review boards since this was a secondary data analysis that lacked personal identifiers. Additionally, NHANES has stringent consent protocols and procedures to ensure the confidentiality and protection of participants from identification. All data analyzed in the present study are publicly available in the Center for Disease Control and Prevention repository https://www.cdc.gov/nchs/nhanes/index.htm (accessed on 10 December 2023).

### 2.2. Dietary Intake

Dietary intakes from foods only (dietary supplement intakes were not included) were estimated using 2 days of 24 h dietary recall interviews that were administered by automated, multiple-pass (AMPM) method [19]. A trained dietary interviewer collected detailed information on all foods and beverages consumed by respondents in the previous 24 h period (midnight to midnight), which was followed by a second dietary recall phone interview for most subjects 3 to 10 days after the first dietary interview as part of the NHANES examination. USDA Food and Nutrient Database for Dietary Studies (FNDDS) was used to determine nutrient intakes for each NHANES cycle [20].

### 2.3. Beef Intake

Beef intakes were assessed using methods previously reported [16] and briefly summarized here. First, we used the FNDDS food codes to determine the amount of beef contained in survey foods and the NHANES cycle-specific USDA Food Patterns Equivalents Database (FPED), which also includes the Food Patterns Equivalents Ingredient Database (FPID) to perform recipe calculations for beef items used as ingredients [20]. The FPID descriptions were examined to determine the proportion of beef in the ingredient: 100% if entirely beef, 50% or 33% if the description indicated one or two other meat types in addition to beef, respectively. For some FNDDS food codes that contained ingredients with missing FPID or food codes for beef, the food code ingredient profile was modified either by using a food code from another NHANES cycle or by using another ingredient code with a similar description. Fresh beef and processed beef were defined by using “pf_meat” and “pf_curedmeat” FPED components, and ground beef was determined based on the ingredient description of beef containing ingredients (i.e., ground beef or similar term) [21]. Beef included fresh, ground, and processed beef. Consumers were defined as those older adults age 60+ years who consumed any amount of beef on either of the two days of dietary recalls, and non-consumers were those who did not consume any amount of beef on either of the two days of dietary recalls.

### 2.4. Statistics

We used SAS 9.4 (SAS Institute, Cary, NC, USA) software for all statistical analyses, and we used two days of dietary weights, strata, and primary sampling units (PSU) to adjust the data for the complex sampling design of NHANES and to provide national representative results. The National Cancer Institute (NCI) method was used to estimate the distributions of usual nutrient intakes [22], and the cut-point method (except for iron, where we used the probability method) was used to estimate the percentage population below the estimated average requirement (EAR) or above the adequate intake (AI) [23]. Data are presented as mean ± standard error; differences between non-consumers and consumers were assessed using *t*-tests with *p* < 0.05 deemed significant.

## 3. Results

### 3.1. Beef Intake

About 68% of older adults age 60+ years were beef consumers with a mean intake of 56.1 ± 1.3 g/day (1.98 oz/day) among consumers; mean per capita beef intake was 38.3 ± 1.1 g/day (1.35 oz/day), which has remained unchanged (P_linear trend_ > 0.05) over the last 16 years among older adults in the US, as previously reported [24].

### 3.2. Demographics

The demographic characteristics of older adults age 60+ years participating in NHANES 2011–2018 are presented in Table 1. The mean age of older adult participants was about 70 years, and almost half were male. More than three-quarters of participants were non-Hispanic white. More than two-thirds of older adults had a household income poverty income ratio (PIR) above 1.85. More than a third of participants had a high school education, slightly less than a third of participants had some post-high school education, and slightly less than a third of participants had at least a Bachelor’s degree. Over 40% of older adults were moderately active, 26% were vigorously active, and 31% were sedentary (based on responses to a physical activity questionnaire). About half of the participants never smoked, and over three-quarters of the participants were overweight or obese (Table 1). There were only a few sporadic and small in magnitude demographic differences between beef non-consumers and consumers. Beef consumers were more likely to be male, non-Hispanic White, and current smokers, and less likely to be Hispanic, non-Hispanic Black, non-Hispanic Asian, have a household income of PIR < 1.35, and non-smokers.

### 3.3. Nutrient Intakes and Adequacy

The current (2011–2018) usual intakes of nutrients and percentage below the EAR or above the AI among older adults age 60+ years are presented in Table 2. Nutrient inadequacy prevalence was high among older adults with over three-quarters of older adults with intakes below the EAR for vitamin D (96%) and vitamin E (84%); over half of older adults had intakes below the EAR for calcium (63%) and magnesium (60%); over a third of older adults had intakes below the EAR for vitamin C (46%) and vitamin A (39%); about 20% of older adults had intakes below the EAR for zinc (21%) and vitamin B_6_ (19%); and more than 10% of older adults had intakes below the EAR for folate (15%). Additionally, only about 30% of older adults were meeting the AI for potassium, and less than 5% were meeting the AI for choline. Almost all older adults had intakes above the AI for sodium.

### 3.4. Association of Beef Intake with Nutrient Intake and Adequacy

Beef intake among older adult consumers was associated with higher usual intakes of energy (+13%), protein (+12%), and several key vitamins and minerals (Table 3). Older adult consumers of beef had higher intakes of calcium (+11%), iron (+11%), phosphorus (+7%), selenium (+10%), zinc (+27%), thiamine (+7%), riboflavin (+9%), niacin (+8%), vitamin B_12_ (+23%), and choline (+8%) than the non-consumers. However, consumers also had higher intakes of sodium (+17%) and lower intakes of vitamin C (−10%) than non-consumers (Table 3).

**Figure 1:**
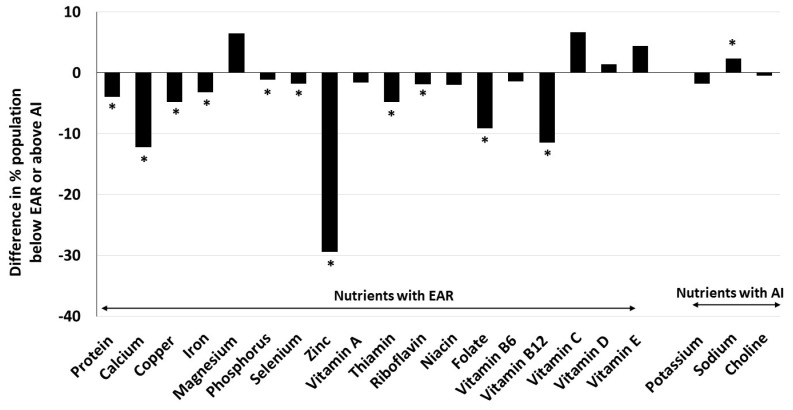
Difference in percent older adults age 60+ years beef consumers and non-consumers with usual intakes below estimated average requirement (EAR) or above adequate intake (AI). Beef consumers were those older adults who consumed any amount of beef on either of the two days of dietary recalls, and non-consumers were those who did not. * significant difference from non-consumers at *p* < 0.05.

Consequently, a greater proportion of older adult beef consumers met recommendations for protein (+4% units), calcium (+12% units), copper (+5% units), zinc (+29% units), thiamin (+5% units), folate (+9% units) and vitamin B_12_ (+11% units). Smaller, but statistically significant, differences among proportions of beef consumers and non-consumers meeting recommendations were also found for iron (+3% units), phosphorus (+1% unit), selenium (+2% units), and riboflavin (+2% units). However, a greater proportion of beef consumers exceeded the AI for sodium (+2% units) and failed to meet nutrient recommendations for magnesium (7% units) and vitamin C (7% units) compared to non-consumers (Table 3; Figure 1).

### 3.5. Intake of Different Beef Types

About 60%, 39%, and 24% of older adults age 60+ years were consumers of fresh beef, ground beef, and processed beef, respectively, and their mean intakes were 54.7 ± 1.5 g/day, 40.8 ± 1.3 g/day, and 23.6 ± 1.0 g/day (1.93 oz/day, 1.44 oz/day, and 0.83 oz/day), respectively. Mean per capita intakes of fresh beef, ground beef, and processed beef were 32.7 ± 1.2 g/day, 16.1 ± 0.7 g/day, and 5.62 ± 0.35 g/day (1.15 oz/day, 0.57 oz/day, and 0.20 oz/day), respectively. The per capita mean intake of different types of beef has remained unchanged (P_linear trend_ > 0.05) over the last 16 years among older adults in the US, as previously reported [24].

### 3.6. Effect of Different Beef Types Intake on Nutrient Intake and Adequacy

Consumers of fresh beef had higher intakes of energy (+11%), protein (+12%), calcium (+8%), iron (+12%), phosphorus (+7%), potassium (+6%), selenium (+10%), sodium (+14%), zinc (+29%), thiamin (+7%), riboflavin (+11%), niacin (+9%), vitamin B_12_ (+25%), and choline (+10%) than non-consumers. A higher percentage of fresh beef consumers also met nutrient recommendations for protein (+4% units), calcium (+9% units), copper (+6% units), iron (+3% units), phosphorus (+1% unit), selenium (+2% units), sodium (+2% units exceeding AI), zinc (+33% units), thiamin (+5% units), riboflavin (+2% units), niacin (+3% units), folate (+10% units), and vitamin B_12_ (11% units) compared to non-consumers (Table 4).

Consumers of ground beef also had higher intakes of energy (+14%), protein (+10%), calcium (+14%), copper (+8%), iron (+13%), magnesium (+5%), phosphorus (+9%), potassium (+7%), selenium (+9%), sodium (+15%), zinc (+24%), thiamin (+10%), riboflavin (+11%), niacin (+10%), folate (+9%), vitamin B_12_ (+22%) and choline (+7%); and a higher percentage of ground beef consumers met recommendations for protein (+2% units), calcium (+14% units), copper (+6% units), iron (+2% units), phosphorus (+1% unit), potassium (+5% units), selenium (+1% unit), sodium (+2% units exceeding AI), zinc (+24% units), thiamin (+6% units), riboflavin (+2% units), niacin (+2% units), folate (+11% units) and vitamin B_12_ (+6% units) compared to non-consumers (Table 5).

Similarly, consumers of processed beef had higher intakes of energy (+11%), protein (+6%), calcium (+8%), iron (+8%), phosphorus (+5%), selenium (+10%), sodium (+17%), zinc (+8%), thiamin (+7%), riboflavin (+6%), vitamin B_12_ (+12%), and choline (+6%); and lower intake of vitamin C (−14%) than non-consumers. A higher percentage of processed beef consumers also met recommendations for protein (+2% units), calcium (+9% units), iron (+1% unit), selenium (+1% unit), sodium (+2% units exceeding AI), zinc (+10% units), and vitamin B_12_ (+5% units) while a lower proportion met nutrient recommendations for magnesium (−9% units), potassium (−7% units), vitamin C (−13% units), and vitamin D (−3% units) compared to non-consumers (Table 6).

## 4. Discussion

This is probably the first report to investigate the association between beef intake and nutrient adequacy in a nationally representative population of American older adults age 60+ years. The results of the present analysis of NHANES cross-sectional data indicate that older adult beef consumers have higher intakes and lower prevalence of inadequacies of key micronutrients, including many “under-consumed nutrients” and “nutrients of public health concern” compared to beef non-consumers. Results for most nutrients are also similar for fresh beef and ground beef consumers.

We found a high prevalence of not meeting nutrient recommendations (estimated as a large percentage of the population below the EAR or a small percentage of the population above the AI) for choline, vitamin D, vitamin E, potassium, calcium, and magnesium among the majority of older adults. We also found inadequate intakes of vitamin C, vitamin A, zinc, vitamin B_6_, and folate in the present cross-sectional analysis of dietary intakes of 5868 older adults who participated in NHANES 2011–2018 (representing about 64 million American older adults). Others have also found similar high prevalence of nutritional inadequacies for several vitamins and minerals among older adults [4,7,25,26,27,28]. Several of these micronutrients mentioned above have been associated with impaired immune function, muscle strength, and physical performance in older adults [29,30]. Older adults generally have lower calorie needs and tend to eat less, but their nutrient needs are similar or even higher than younger adults. Therefore, maintaining a nutrient-dense diet is critically important for them.

In the present analysis, the older adult consumers of beef had higher intakes of energy, protein, calcium, iron, phosphorus, selenium, zinc, thiamin, riboflavin, niacin, vitamin B_12_, and choline. Calcium is a nutrient of concern for all population groups, including older adults, and protein and vitamin B_12_ are nutrients of special consideration for older adults [8]. Although energy intake among both consumers and non-consumers of beef was below or at the lower side of recommended energy intake for moderately active older adults [8], the 13% higher energy intake (2008 kcal/day vs. 1775 kcal/day) among consumers than non-consumers suggests that beef consumption may help older adults to maintain calorie intake.

Beef consumers also had a lower inadequacy prevalence for calcium, copper, iron, phosphorus, selenium, zinc, thiamin, riboflavin, niacin, folate, and vitamin B_12_, but a higher prevalence of inadequacy for magnesium and vitamin C compared to non-consumers. While the observed differences in the percent population not meeting nutrient recommendations (% population below the EAR) between older adult beef consumers and non-consumers for zinc, calcium, and vitamin B_12_ were more than 10% units, the differences for other nutrients were in mid-single digits (see Table 3). Since we used population-weighted nationally representative data, a sample size of 5868 represented 63.6 million older adults, of which 20.2 million (sample size 2072) were non-consumers and 43.5 million (sample size 3796) were consumers of beef and a 1% unit change in prevalence of meeting nutritional requirement among consumers would translate to 0.435 million (435 thousand) older adults. The results of a decrease in proportion of older adult population below the EAR for zinc from 41.5% among non-consumers to 12.1% among beef consumers suggest that about 5.94 million older adult non-consumers would no longer be below the EAR for zinc by incorporating beef into their diet and consuming a food pattern similar to beef consumers in this study [calculated as 29.4% (difference in consumer and non-consumers for nutrient adequacy for zinc) times 20.2 million (the population of non-consumers)]. Similarly, we also estimated that by incorporating beef into their diets and consuming an overall diet similar to that of beef consumers in this study, about 0.81, 2.46, 0.97, 0.98, 0.39, 1.84 and 2.31 million older adult non-consumers would potentially be at or above the EAR for intakes of protein, calcium, copper, thiamin, riboflavin, folate, and vitamin B_12_, respectively.

Interestingly, while beef consumers had significantly higher intakes of choline than non-consumers, the difference in the prevalence of the proportion of the population above the AI did not reach statistical significance. Similarly, beef consumers had a lower prevalence of inadequacy of folate than non-consumers, while the differences in their intakes did not reach statistical significance.

We could not find any other studies examining nutrient intake or adequacy (percentage below the EAR/above the AI) among older adults separated by beef consumption. Although beef has been shown to contribute a significant amount of nutrients in the diets of American adults [13,16] and its consumption was associated with higher nutrient intakes and adequacies in some US population groups [14,15,31], none of these studies specifically analyzed beef intake and nutrition adequacy among a population of older adults age 60+ years. A cross-sectional study of an older adult population did not find any association between beef consumption and zinc, vitamin B_12_, or iron concentration in the blood [32]. However, this study did not analyze the nutrient intake or nutrient adequacy.

Consumers of different types of beef (fresh beef, ground beef, and processed beef) also generally had higher intakes of energy, protein, calcium, phosphorus, selenium, sodium, zinc, thiamin, riboflavin, vitamin B_12_, and choline than their respective non-consumers. A higher prevalence of meeting nutrient recommendations for protein, calcium, copper, iron, phosphorus, potassium, selenium, zinc, thiamin, riboflavin, niacin, folate, and vitamin B_12_ was also noted among consumers of fresh, ground, and processed beef than their respective non-consumers. However, the differences in prevalence above the AI for potassium for fresh beef and for copper, phosphorus, thiamin, riboflavin, and niacin for processed beef were not statistically significant in consumers of processed beef. Additionally, processed beef consumers had a lower prevalence of adequacy for magnesium, vitamin C, and vitamin D compared to non-consumers. This is also the first study that examined the intake of different types of beef (fresh beef, ground beef, and processed beef) in relation to their contribution to nutrient adequacy in a nationally representative US older adult population. In an analysis of NHANES 2011–2018, we recently reported that beef (including lean fresh beef, ground beef, and processed beef) contributes to energy, protein, and several key micronutrients in the diet of American adults [16].

It is interesting to note that beef intake was associated with higher intakes and lower prevalence of inadequacy of calcium in older adults in the current study. This is probably due to the overall dietary pattern, possible differences in intake of food groups, and likely more dairy consumption, since beef is generally not considered a source of calcium and only contributes a small amount in the diet [16]. Regardless of beef being a part of diets, there is still considerable opportunity for improvement of nutrient intake to decrease nutrient inadequacy in this population.

The major strengths of our study included the use of a large nationally representative, population-based sample achieved through combining several years of NHANES data sets and the use of the NCI method to assess usual intake and the percentage of the population below the EAR or above the AI. Additionally, we used previously published methods for determining foods containing beef and, therefore, beef consumers [16]. The present study also analyzed the association of different beef types (fresh, ground, and processed) with nutrient intake and adequacy. A major limitation of the current study, as with any cross-sectional study, is the inability to determine a cause-and-effect relationship or the long-term dietary effects. There is a potential for bias in the use of memory-based self-reported dietary recalls [33]. Although 24 h dietary recalls collected on two different days were utilized in this study, there is a possibility that participants consumed beef on other days, which would result in underestimating the mean intake of beef and beef consumers. Associations between beef intake and nutrient intakes or adequacies, as noted in the present analysis, may also be due to the differences in the intake of other foods. Additionally, we assessed the nutrient intakes from foods only and did not include intakes from dietary supplements.

## 5. Conclusions

The results show that beef consumption was associated with higher nutrient intake and a greater proportion of those meeting nutrient recommendations in US older adults for certain key nutrients, including some “nutrients of public health concern”, suggesting that beef may play a vital role in reducing the incidence of under-nutrition in this population. Additionally, the intakes of different beef types (fresh, ground, and processed) were also associated with higher nutrient intakes and prevalence of adequacy for several nutrients. The results suggest that care should be taken to ensure that the dietary recommendations to limit or reduce beef, for any reason, can replace nutrients that could be expected to be provided by beef (e.g., iron, phosphorus, choline, protein, zinc, and vitamin B_12_) in older adults. The long-term impact of beef consumption on diet quality, nutrient intake, and health promotion needs to be examined in future studies.

## Figures and Tables

**Table 1:** Demographics of older adults age 60+ years associated with beef consumption, NHANES 2011–2018 data.

	Total Population	Beef		
		Non-Consumers	Consumers	*p* Value
Sample N	5868	2072	3796	
Population N	63,614,931	20,160,054	43,454,877	
Age (mean)	69.6 ± 0.2	70.0 ± 0.3	69.4 ± 0.2	0.1011
Gender (% Male)	46.4 ± 0.8	38.8 ± 1.5	49.9 ± 1.3	<0.0001
Ethnicity				
Hispanic (%)	7.95 ± 0.87	9.37 ± 1.04	7.29 ± 0.92	0.0203
Non-Hispanic White (%)	77.6 ± 1.5	72.1 ± 2.1	80.2 ± 1.6	<0.0001
Non-Hispanic Black (%)	8.49 ± 0.86	10.16 ± 1.11	7.71 ± 0.86	0.0073
Non-Hispanic Asian (%)	3.92 ± 0.41	6.72 ± 0.84	2.63 ± 0.33	<0.0001
Other (%)	2.00 ± 0.28	1.64 ± 0.33	2.16 ± 0.40	0.3410
Poverty Income Ratio (PIR)				
<1.35 (%)	17.4 ± 1.0	20.4 ± 1.5	16.1 ± 1.0	0.0033
1.35 ≤ 1.85 (%)	11.1 ± 0.9	10.2 ± 0.9	11.4 ± 1.1	0.3171
>1.85 (%)	71.5 ± 1.5	69.4 ± 1.9	72.5 ± 1.7	0.1112
Education				
≤High School (%)	38.3 ± 1.3	38.0 ± 1.5	38.5 ± 1.5	0.7879
Some post-high school education (%)	29.8 ± 1.3	29.0 ± 1.7	30.2 ± 1.4	0.5153
≥Bachelor’s degree (%)	31.9 ± 1.7	33.0 ± 1.9	31.4 ± 2.1	0.4636
Physical Activity				
Sedentary (%)	30.8 ± 1.1	31.7 ± 1.7	30.5 ± 1.3	0.5373
Moderate (%)	43.6 ± 1.2	43.5 ± 1.9	43.7 ± 1.4	0.9392
Vigorous (%)	25.5 ± 1.1	24.8 ± 1.6	25.8 ± 1.3	0.6037
Smoking Never (%)	49.8 ± 1.1	53.3 ± 1.8	48.2 ± 1.2	0.0125
Smoking Current (%)	11.6 ± 0.6	9.7 ± 1.0	12.5 ± 0.7	0.0244
Overweight (%)	35.4 ± 1.2	33.0 ± 1.7	36.5 ± 1.4	0.1046
Obese (%)	40.5 ± 1.4	39.2 ± 2.1	41.1 ± 1.6	0.4506

Beef consumers were those older adults who consumed any amount of beef on either of the two days of dietary recalls, and non-consumers were those who did not. Data are presented as mean ± standard error.

**Table 2:** Usual intakes of nutrients and percent of older adults age 60+ years (n = 5868) meeting nutrient recommendations, NHANES 2011–2018 data.

	Usual Intake	% Below EAR or Above AI
Energy (kcal)	1936 ± 15	
EAR nutrients		
Protein (g)	74.2 ± 0.7	2.68 ± 0.43
Calcium (mg)	877 ± 12	63.0 ± 1.6
Copper (mg)	1.23 ± 0.01	7.22 ± 0.65
Iron (mg)	14.2 ± 0.1	1.08 ± 0.23
Magnesium (mg)	291 ± 3	60.2 ± 1.3
Phosphorus (mg)	1277 ± 13	1.20 ± 0.27
Selenium (µg)	104 ± 1	0.85 ± 0.20
Zinc (mg)	10.4 ± 0.1	21.5 ± 1.6
Vitamin A, RAE (µg)	668 ± 14	38.9 ± 2.4
Thiamin (mg)	1.53 ± 0.02	7.89 ± 0.85
Riboflavin (mg)	2.04 ± 0.02	2.94 ± 0.41
Niacin (mg)	23.0 ± 0.3	1.89 ± 0.43
Folate, DFE (µg)	496 ± 6	15.2 ± 1.2
Vitamin B_6_ (mg)	1.92 ± 0.03	19.2 ± 1.5
Vitamin B_12_ (µg)	4.67 ± 0.10	5.59 ± 0.85
Vitamin C (mg)	80.8 ± 1.8	46.2 ± 1.6
Vitamin D (µg)	4.66 ± 0.11	95.6 ± 0.7
Vitamin E, ATE (mg)	8.72 ± 0.15	84.4 ± 1.3
AI nutrients		
Potassium (mg)	2626 ± 23	29.9 ± 1.2
Sodium (mg)	3157 ± 28	98.5 ± 0.3
Choline (mg)	316 ± 3	3.78 ± 0.61

Two days 24 h dietary recall data. Data are presented as mean ± standard error. % below the EAR for protein was based on g/kg of ideal body weight. AI: adequate intake; ATE: alpha-tocopherol equivalents; EAR: estimated average requirement; DFE: dietary folate equivalents; RAE: retinol activity equivalents.

**Table 3:** Usual nutrient intakes and percentage of population meeting nutrient recommendations among older adults age 60+ years non-consumers and consumers of beef, NHANES 2011–2018 data.

	Usual Intakes			% Meeting Recommendations		
	Beef Non-Consumers	Beef Consumers	*p* Value	Beef Non-Consumers	Beef Consumers	*p* Value
Energy (kcal)	1775 ± 30	2008 ± 22	<0.0001			
EAR nutrients				% Below EAR		
Protein (g)	68.7 ± 1.4	76.7 ± 0.9	<0.0001	5.34 ± 1.09	1.34 ± 0.45	0.0007
Calcium (mg)	813 ± 18	905 ± 15	0.0001	71.3 ± 2.0	59.1 ± 2.1	<0.0001
Copper (mg)	1.21 ± 0.03	1.23 ± 0.02	0.5427	10.4 ± 1.3	5.61 ± 0.87	0.0016
Iron (mg)	13.2 ± 0.3	14.6 ± 0.2	<0.0001	3.42 ± 0.63	<1.00	<0.0001
Magnesium (mg)	294 ± 6	289 ± 4	0.5003	56.1 ± 2.4	62.6 ± 1.6	0.0236
Phosphorus (mg)	1218 ± 22	1302 ± 15	0.0019	1.99 ± 0.43	<1.00	0.0444
Selenium (µg)	97.5 ± 2.1	107 ± 2	0.0002	2.10 ± 0.53	<1.00	0.0015
Zinc (mg)	8.82 ± 0.21	11.2 ± 0.1	<0.0001	41.5 ± 2.3	12.1 ± 1.9	<0.0001
Vitamin A, RAE (µg)	662 ± 19	670 ± 17	0.7599	40.1 ± 2.9	38.5 ± 3.2	0.7120
Thiamin (mg)	1.46 ± 0.03	1.56 ± 0.02	0.0169	11.3 ± 1.6	6.45 ± 1.08	0.0135
Riboflavin (mg)	1.92 ± 0.04	2.10 ± 0.03	0.0001	4.31 ± 0.69	2.38 ± 0.50	0.0227
Niacin (mg)	21.8 ± 0.7	23.6 ± 0.3	0.0174	3.22 ± 1.04	1.20 ± 0.47	0.0765
Folate, DFE (µg)	488 ± 10	499 ± 8	0.4247	21.3 ± 1.8	12.2 ± 1.4	0.0001
Vitamin B_6_ (mg)	1.92 ± 0.08	1.92 ± 0.04	0.9565	20.4 ± 3.2	19.0 ± 1.8	0.6847
Vitamin B_12_ (µg)	4.03 ± 0.14	4.97 ± 0.13	<0.0001	13.4 ± 2.1	1.94 ± 0.76	<0.0001
Vitamin C (mg)	86.3 ± 3.2	78.0 ± 2.3	0.0340	41.9 ± 2.8	48.6 ± 2.0	0.0489
Vitamin D (µg)	4.93 ± 0.15	4.54 ± 0.14	0.0557	94.6 ± 0.9	96.0 ± 0.9	0.2916
Vitamin E, ATE (mg)	8.72 ± 0.24	8.71 ± 0.19	0.9853	81.5 ± 1.8	85.9 ± 1.8	0.0834
AI nutrients				% Above AI		
Potassium (mg)	2568 ± 47	2656 ± 28	0.1065	31.3 ± 2.2	29.5 ± 1.5	0.5117
Sodium (mg)	2831 ± 51	3310 ± 38	<0.0001	96.9 ± 0.8	99.2 ± 0.3	0.0062
Choline (mg)	300 ± 6	324 ± 4	0.0010	4.23 ± 1.16	3.71 ± 0.70	0.6965

Beef consumers were those older adults who consumed any amount of beef on either of the two days of dietary recalls, and non-consumers were those who did not. Data are presented as mean ± standard error. % below the EAR for protein were based on g/kg of ideal body weight. AI: adequate intake; ATE: alpha-tocopherol equivalents; EAR: estimated average requirement; DFE: dietary folate equivalents; RAE: retinol activity equivalents.

**Table 4:** Usual nutrient intakes and percentage of population meeting nutrient recommendations among older adults age 60+ years non-consumers and consumers of fresh beef, NHANES 2011–2018 data.

	Usual Intakes			% Meeting Recommendations		
	Fresh Beef Non-Consumers	Fresh Beef Consumers	*p* Value	Fresh Beef Non-Consumers	Fresh Beef Consumers	*p* Value
Energy (kcal)	1812 ± 28	2017 ± 23	<0.0001			
EAR nutrients				% Below EAR		
Protein (g)	69.2 ± 1.3	77.5 ± 0.9	<0.0001	4.96 ± 0.96	1.00 ± 0.36	0.0001
Calcium (mg)	835 ± 17	904 ± 16	0.0032	68.8 ± 1.9	59.3 ± 2.3	0.0017
Copper (mg)	1.19 ± 0.02	1.25 ± 0.02	0.0865	10.8 ± 1.1	4.87 ± 0.92	<0.0001
Iron (mg)	13.2 ± 0.2	14.8 ± 0.2	<0.0001	2.83 ± 0.47	<1.00	<0.0001
Magnesium (mg)	288 ± 5	292 ± 4	0.5622	58.5 ± 2.0	61.5 ± 1.9	0.2655
Phosphorus (mg)	1226 ± 21	1308 ± 16	0.0020	1.95 ± 0.47	<1.00	0.0170
Selenium (µg)	98.6 ± 1.9	108 ± 2	0.0003	1.89 ± 0.54	<1.00	0.0022
Zinc (mg)	8.87 ± 0.17	11.5 ± 0.2	<0.0001	40.8 ± 2.0	7.99 ± 1.90	<0.0001
Vitamin A, RAE (µg)	665 ± 16	670 ± 18	0.8359	39.4 ± 2.7	39.1 ± 3.2	0.9465
Thiamin (mg)	1.47 ± 0.03	1.57 ± 0.03	0.0091	10.9 ± 1.4	5.80 ± 1.05	0.0038
Riboflavin (mg)	1.92 ± 0.03	2.13 ± 0.03	<0.0001	4.29 ± 0.68	1.96 ± 0.49	0.0055
Niacin (mg)	21.9 ± 0.6	23.8 ± 0.4	0.0042	3.48 ± 1.01	<1.00	0.0127
Folate, DFE (µg)	485 ± 9	503 ± 9	0.1539	20.8 ± 1.8	11.2 ± 1.4	<0.0001
Vitamin B_6_ (mg)	1.87 ± 0.07	1.95 ± 0.04	0.2749	21.8 ± 3.1	17.0 ± 1.6	0.1704
Vitamin B_12_ (µg)	4.05 ± 0.11	5.08 ± 0.15	<0.0001	12.1 ± 1.6	1.41 ± 0.69	<0.0001
Vitamin C (mg)	83.7 ± 2.8	79.0 ± 2.4	0.2069	44.2 ± 2.5	47.4 ± 2.0	0.3094
Vitamin D (µg)	4.84 ± 0.14	4.55 ± 0.15	0.1467	95.4 ± 0.9	95.7 ± 1.0	0.8196
Vitamin E, ATE (mg)	8.63 ± 0.21	8.76 ± 0.21	0.6730	82.5 ± 1.6	85.7 ± 2.0	0.2184
AI nutrients				% Above AI		
Potassium (mg)	2540 ± 40	2690 ± 31	0.0032	29.4 ± 1.8	30.7 ± 1.7	0.6006
Sodium (mg)	2914 ± 48	3321 ± 42	<0.0001	97.3 ± 0.7	99.2 ± 0.3	0.0070
Choline (mg)	297 ± 5	328 ± 4	<0.0001	3.25 ± 0.78	4.03 ± 0.85	0.4974

Fresh beef consumers were those older adults who consumed any amount of fresh beef on either of the two days of dietary recalls, and non-consumers were those who did not. Data are presented as mean ± standard error. % below the EAR for protein were based on g/kg of ideal body weight. AI: adequate intake; ATE: alpha-tocopherol equivalents; EAR: estimated average requirement; DFE: dietary folate equivalents; RAE: retinol activity equivalents.

**Table 5:** Usual nutrient intakes and percentage of population meeting nutrient recommendations among older adults age 60+ year non-consumers and consumers of ground beef, NHANES 2011–2018 data.

	Usual Intakes			% Meeting Recommendations		
	Ground Beef Non-Consumers	Ground Beef Consumers	*p* Value	Ground Beef Non-Consumers	Ground Beef Consumers	*p* Value
Energy (kcal)	1836 ± 23	2087 ± 31	<0.0001			
EAR nutrients				% Below EAR		
Protein (gm)	71.3 ± 1.0	78.6 ± 1.1	<0.0001	3.03 ± 0.72	1.14 ± 0.39	0.0205
Calcium (mg)	831 ± 14	944 ± 19	<0.0001	68.6 ± 1.5	54.7 ± 3.1	0.0001
Copper (mg)	1.19 ± 0.02	1.28 ± 0.03	0.0039	9.41 ± 0.92	4.32 ± 0.82	<0.0001
Iron (mg)	13.5 ± 0.2	15.3 ± 0.3	<0.0001	1.79 ± 0.38	<1.00	<0.0001
Magnesium (mg)	285 ± 4	299 ± 5	0.0244	61.0 ± 1.6	58.0 ± 2.2	0.2721
Phosphorus (mg)	1234 ± 16	1341 ± 19	<0.0001	1.64 ± 0.41	<1.00	0.0138
Selenium (mcg)	101 ± 1	110 ± 2	0.0004	1.15 ± 0.40	<1.00	0.0238
Zinc (mg)	9.54 ± 0.13	11.8 ± 0.2	<0.0001	32.3 ± 2.0	8.17 ± 2.12	<0.0001
Vitamin A, RAE (µg)	669 ± 14	667 ± 22	0.9468	38.7 ± 2.7	40.4 ± 3.7	0.7108
Thiamin (mg)	1.47 ± 0.02	1.62 ± 0.3	0.0001	10.4 ± 1.1	4.26 ± 0.83	<0.0001
Riboflavin (mg)	1.96 ± 0.02	2.17 ± 0.04	<0.0001	3.49 ± 0.55	1.84 ± 0.51	0.0272
Niacin (mg)	22.2 ± 0.4	24.3 ± 0.4	0.0002	2.72 ± 0.67	<1.00	0.0054
Folate, DFE (µg)	480 ± 7	521 ± 12	0.0028	19.7 ± 1.6	8.31 ± 1.41	<0.0001
Vitamin B_6_ (mg)	1.90 ± 0.04	1.95 ± 0.05	0.4765	20.3 ± 2.2	17.2 ± 2.2	0.3272
Vitamin B_12_ (mcg)	4.31 ± 0.10	5.25 ± 0.18	<0.0001	7.98 ± 1.44	1.49 ± 0.61	<0.0001
Vitamin C (mg)	81.4 ± 2.2	79.8 ± 2.9	0.6566	45.3 ± 1.9	47.7 ± 2.4	0.4382
Vitamin D (µg)	4.74 ± 0.15	4.54 ± 0.15	0.3240	95.4 ± 1.0	96.1 ± 1.0	0.6296
Vitamin E, ATE (mg)	8.46 ± 0.17	9.07 ± 0.26	0.0524	85.1 ± 1.4	82.8 ± 2.5	0.4225
AI nutrients				% Above AI		
Potassium (mg)	2561 ± 30	2732 ± 35	0.0002	28.4 ± 1.6	33.1 ± 1.8	0.0462
Sodium (mg)	2975 ± 34	3433 ± 56	<0.0001	97.8 ± 0.5	99.5 ± 0.2	0.0007
Choline (mg)	307 ± 4	330 ± 6	0.0012	3.26 ± 0.70	4.66 ± 1.12	0.2895

Ground beef consumers were those older adults who consumed any amount of ground beef on either of the two days of dietary recalls, and non-consumers were those who did not. Data are presented as mean ± standard error. % below the EAR for protein were based on g/kg of ideal body weight. AI: adequate intake; ATE: alpha-tocopherol equivalents; EAR: estimated average requirement; DFE: dietary folate equivalents; RAE: retinol activity equivalents.

**Table 6:** Usual nutrient intakes and percentage of population meeting nutrient recommendations among older adults age 60+ year non-consumers and consumers of processed beef, NHANES 2011–2018 data.

	Usual Intakes			% Meeting Recommendations		
	Processed Beef Non-Consumers	Processed Beef Consumers	*p* Value	Processed Beef Non-Consumers	Processed Beef Consumers	*p* Value
Energy (kcal)	1887 ± 17	2094 ± 37	<0.0001			
EAR nutrients				% Below EAR		
Protein (g)	73.1 ± 0.9	77.6 ± 1.6	0.0138	3.02 ± 0.51	1.02 ± 0.62	0.0129
Calcium (mg)	860 ± 15	925 ± 23	0.0178	65.4 ± 1.8	56.1 ± 3.0	0.0089
Copper (mg)	1.22 ± 0.02	1.25 ± 0.04	0.4341	7.54 ± 0.79	6.54 ± 1.43	0.5384
Iron (mg)	13.9 ± 0.2	15.0 ± 0.3	0.0025	1.09 ± 0.27	<1.00	0.0223
Magnesium (mg)	293 ± 4	283 ± 7	0.1997	58.0 ± 1.6	67.0 ± 2.7	0.0041
Phosphorus (mg)	1261 ± 15	1323 ± 2.8	0.0495	1.35 ± 0.28	<1.00	0.1154
Selenium (µg)	102 ± 1	112 ± 3	0.0016	1.03 ± 0.28	<1.00	0.0032
Zinc (mg)	10.2 ± 0.1	11.1 ± 0.2	0.0013	24.7 ± 1.6	15.2 ± 3.4	0.0109
Vitamin A, RAE (µg)	669 ± 14	671 ± 35	0.9388	39.5 ± 2.4	36.9 ± 6.6	0.7085
Thiamin (mg)	1.51 ± 0.02	1.60 ± 0.5	0.0463	8.70 ± 0.93	5.64 ± 1.79	0.1294
Riboflavin (mg)	2.02 ± 0.002	2.13 ± 0.05	0.0359	3.19 ± 0.49	2.29 ± 0.68	0.2827
Niacin (mg)	22.7 ± 0.4	24.0 ± 0.7	0.0867	1.87 ± 0.45	1.76 ± 0.95	0.9200
Folate, DFE (µg)	491 ± 7	514 ± 16	0.1868	16.4 ± 1.3	11.9 ± 2.3	0.0851
Vitamin B_6_ (mg)	1.94 ± 0.04	1.85 ± 0.06	0.1700	18.7 ± 1.7	21.1 ± 4.1	0.5952
Vitamin B_12_ (µg)	4.54 ± 0.12	5.09 ± 0.23	0.0368	6.62 ± 1.22	1.93 ± 1.01	0.0030
Vitamin C (mg)	83.7 ± 2.1	72.0 ± 3.8	0.0065	43.1 ± 1.8	56.3 ± 3.1	0.0002
Vitamin D (µg)	4.76 ± 0.14	4.36 ± 0.22	0.1194	94.8 ± 0.9	98.3 ± 1.2	0.0169
Vitamin E, ATE (mg)	8.67 ± 0.17	8.88 ± 0.29	0.5221	83.9 ± 1.4	84.4 ± 3.0	0.8716
AI nutrients				% Above AI		
Potassium (mg)	2635 ± 29	2618 ± 51	0.7648	31.7 ± 1.4	25.0 ± 2.4	0.0180
Sodium (mg)	3036 ± 33	3553 ± 86	<0.0001	98.0 ± 0.4	99.8 ± 0.2	<0.0001
Choline (mg)	311 ± 4	330 ± 8	0.0322	3.97 ± 0.71	2.95 ± 1.28	0.4898

Processed beef consumers were those older adults who consumed any amount of processed beef on either of the two days of dietary recalls, and non-consumers were those who did not. Data are presented as mean ± standard error. % below the EAR for protein were based on g/kg of ideal body weight. AI: adequate intake; ATE: alpha-tocopherol equivalents; EAR: estimated average requirement; DFE: dietary folate equivalents; RAE: retinol activity equivalents.

## Data Availability

The datasets analyzed in this study are available in the Center for Disease Control and Prevention repository; available online: http://www.cdc.gov/nchs/nhanes/ (accessed on 10 December 2023).
